# A safe bridge – parents’ and staff’s experiences of an antenatal visit introducing a home visiting program in disadvantaged areas

**DOI:** 10.1186/s12913-025-13578-9

**Published:** 2025-10-22

**Authors:** Gunilla Lönnberg, Jennifer Leissner, Georgina Warner, Anna Sarkadi

**Affiliations:** Department of Public Health and Caring Sciences, Uppsala University, Uppsala, Sweden

**Keywords:** Child health services, Home visits, Interprofessional team, Midwife, Child health services nurse, Social worker, Antenatal care

## Abstract

**Aim:**

To explore parents’ and staff’s experiences regarding a novel visit in late pregnancy, involving the midwife, child health nurse, and social services, that had been added to a postpartum extended home visiting program.

**Design:**

Qualitative interview study.

**Methods:**

Semi-structured interviews and focus groups were carried out with twelve parents, ten nurses, eight social workers and nine midwifes. Nine of the participating parents were foreign-born; four of them required a translator. The interviews were recorded and transcribed verbatim and the data was analyzed with thematic network analysis.

**Results:**

The experiences are summarized in the Global Theme: ‘Overcoming challenges and creating a feeling of trust and care for the whole family’ and derived from the Organizing Themes: 1) Fear, stigma and unclear role of social services, 2) Challenges with logistics and roles, 3) Providing/having access to information and emotional support, 4) Getting a head start on the relationship. Both parents and staff noted that the antenatal visit facilitated trust transfer from the midwife to the child health nurse and social worker. Logistics were sometimes challenging without designated coordinators. The visit helped staff identify families’ specific needs and tailor the support accordingly. Getting a head start on the relationship was benefiting future home visits.

**Conclusions:**

Building trust and creating a sense of security are central aspects of introducing a home visiting program and the staff who will provide it during an antenatal visit, led by a midwife.

**Implications for the profession and patient care:**

A novel visit in late pregnancy, introducing the nurse and social worker who will carry out the home visits to the parents before childbirth, is an effective way of initiating a home visiting program, building trust between parents and staff. Adequate resources for logistics and administration are a prerequisite for implementing a common visit between agencies.

**Supplementary Information:**

The online version contains supplementary material available at 10.1186/s12913-025-13578-9.

## Background

Data show that mental and physical ill-health, as well as an increased use of health care is linked to low socioeconomic status in Sweden [1]. To counteract long-term risks of disease into adulthood due to inequalities in health and toxic stress during childhood early interventions are both most desirable and potentially most effective [2, 3]. Economists have also shown an interest in early childhood and emphasize that early childhood is crucial for the “accumulation of health” and human capital, making early childhood worth investing in [4].

This is consistent with Swedish public health policy where the government has promised to close the health gaps within a generation [5]. Furthermore, the Commission for Equal Health has highlighted the important compensatory mission of child care and child health service [6]. This is in line with the concept of proportional universalism coined by Marmot, by which services strive proportionately harder to reach parts of the population with health promotion and prevention programs that they would otherwise not have taken part in, proportionately to their needs [7].

Extended home visiting programs during infancy have been shown to be a useful tool to deliver promotion and prevention interventions [8]. Such programs can be given as a targeted intervention to families with a high risk of ill health or as a preventive universal intervention offered to all families [9]. The popularity of home visiting programs is supported by their potential benefits including convenience for parents in that they do not have to arrange transportation, an opportunity for personalized services and ‘whole family’ engagement, as well as enabling home visitors to observe the family’s living environment, tailor services to address specific needs, and establish relationships in a manner that may not be possible with other types of interventions [10].

Research on parental experiences of support services indicate that parents want a blend of nurturing support and guidance in their parental role. They express a preference for services characterized by empathy, competence, practicality, respect and flexibility [11]. In situations of adversity, parents may fear a loss of autonomy when turning to support services and want non-judgmental support and continuity of care [12, 13].

In Sweden, upon a baby’s birth, the family is entitled to Child Health Service (CHS). This sector is staffed by child health nurses, who connect families with physicians, psychologists, dieticians, social workers, and speech therapists if needed. The overarching aim is to promote child health through health assessments, guidance, and vaccinations for all children, and to provide parental support. Furthermore, the goals encompass monitoring parental mental health, strengthening familial social bonds, and facilitating responsive parenting. Child health nurses initially engage with children and their parents at birth, with families receiving approximately 13 visits over the first six years of their children’s lives. Among these visits, two are conducted at home (the first visit and another at 8 months), while the remainder are held at CHS centers. [14].

With the aim to improve health in children in a disadvantaged area in Stockholm an extended home visiting programme was initiated in 2013 with in total six home visits until the child is 15 months of age. The program is called Rinkeby home visiting program and is performed by a CHS nurse and a parental advisor from the social services. The content of the program covers all the domains of nurturing care as recommended by WHO Commission on Social Determinants of Health [15]. Through the encounters between parents and professionals the program encompasses strengthening of positive parenting, facilitating access to supplementary services, early detection of needs, and offering tailored psychosocial support to each family’s unique circumstances [16]. Qualitative findings show that migrant fathers perceive that the extended home visits increased their parental self-efficacy as well as their knowledge of local recourses and societal services for their family [17]. Furthermore, CHS nurses perceive that conducting team-based visits together with a social worker led to achieving goals, leveraging expertise beyond individual team members’ capacities, delivering superior care quality, and better meeting the needs of children and families compared to non-team-based visits [18].

In 2021, the National Board of Health and Welfare initiated a pilot project in four CHS centers, all situated in disadvantages areas in Swedish cities. Building on the Rinkeby model the extended home visiting program ‘Together for a safe start’ was offered to all families expecting their first child, or their first child born in Sweden, in these sites. Just like The Rinkeby home visiting program, ‘Together for a safe start’ offers six team-based home visits. However, there are also two additional visits (thus a total of eight visits): one antenatal visit in pregnancy week 34–38 which is held at the maternal health center and one when the child is two years of age which takes place at the CHS center. In some areas these centers are co-located in a family center.

The rationale for adding two more visits and starting antenatally is grounded in the literature. Reviews of home visiting programs support a greater number of visits over a longer duration as well as antenatal recruitment, as programs that commence before childbirth show a greater number of significant positive outcomes when compared to studies that start postnatally [10]. A recent Swedish study also supports supplementing the home visiting program with an antenatal visit as it seems to lead to fewer absentees at routine visits for both mothers and children, as well as higher breastfeeding and vaccination rates [19].

## Aim

The aim of this study was to explore parents’ and staff’s experiences of the novel antenatal visit, involving the midwife, child health nurse, and social worker, as part of the extended home visiting program.

## Methods

### Design

This is a qualitative descriptive study [20] using 4 focus groups and 28 individual interviews with midwifes (n = 9), CHS nurses (n = 10), social workers (n = 8) and parents (n = 12).

### Study setting, recruitment and inclusion criteria

The home visiting program ‘Together for a safe start’ was implemented in 2021 and delivered by a multi-professional team from the child health services in collaboration with maternal health care, social services and dental care in disadvantaged areas in four cities. It was offered to families expecting their first child, or their first child born in Sweden. The program is based on ‘The Rinkeby home visit program’ which consists of six home visits from newborn to 15 months of age [21], with the addition of two extra visits: One at the maternal care clinic during late pregnancy and one at the child health care clinic when the child is 2 years of age. During the antenatal visit the expectant parents meet their midwife as well as the social worker and child health services nurse (CHS nurse) who will carry out the home visits once their child is born. Topics such as becoming a parent, access to social support, breastfeeding and child-safety are discussed and the parents are encouraged to raise questions and concerns that matter to them.

Because of different conditions, such as having services co-located or not, and various practical limitations, such as sick leave and vacancies, it was necessary to make some local adjustments regarding the antenatal visit. Additionally, due to the ongoing covid-19 pandemic, further alterations to the original design needed to be made in order to implement the program while ensuring safety and obeying current regulations. Due to the pandemic, alterations were also made in the ordinary maternal health care and child health care programs with consequences such as canceled parent groups and restrictions regarding co-parents at the maternal health visits. Consequently, the antenatal visits were executed differently, which is summarized in Table 1 below:

**Table 1:** Local adjustments of the antenatal visit

Site	A	B	C	D
Location	Family center (co-location of maternal health care, CHS and social services).	Health care center and then CHS next door.	Maternal health care clinic and video call.	Health care center a
Time	30 min with midwife +30 min joined by CHS nurse and social worker.	45 min with midwife +15 min joined by CHS nurse and social worker.	Regular check up with midwife +15–20 min joined by CHS nurse and social worker often via video call.	Regular check up with midwife +5–10 min joined by CHS nurse and social worker often via video call.
Participating parents	Both parents	Both parents	Both parents	Only birthing parent.

Purposive sampling was used with two strategies, one for staff and one for parents. Staff who were providing the home visiting program at the four sites were asked to participate in the study by one of the researchers. In the sites where the team of midwifes and nurses were larger than four persons, they were asked to select those among them who had the most experience of the antenatal visit. The researchers were flexible in scheduling the interviews with the staff to fit their availability. To facilitate for the participating staff with a heavy workload they were given the option to form a focus group of two to three persons of the same profession and from the same clinic.

Parents who received the extended home visiting program in the four sites were asked to participate in the study by their midwife or CHS nurse. The inclusion criteria were that the parents had been to the antenatal visit and that their child was between 1 and 6 months old. The selection of participants was done by midwives and CHS nurses. If all parents meeting these criteria were asked if they wanted to participate in the study is uncertain, and it is also unknown how many parents were asked and declined, and why. The parents were informed that the aim of the study was to understand how parents had experienced the antenatal visit. They were also informed that their involvement would mean being interviewed one-on-one by a researcher about their experiences and that the meeting would take place either in person, by telephone or video call.

### Data collection

Data was collected between May 2021 and October 2021 through 28 semi-structured interviews and 4 focus groups using interview guides developed for this study (Supplementary file 1 and 2). The interviews and focus groups with staff lasted between 25–60 minutes, with an average of 38 minutes, and the interviews with parents lasted between 20–40 minutes, with an average of 28 minutes. All the interviews with staff and eight of the interviews with parents were carried out by the first author, GL. The other four of the interviews with parents were carried out by author JL. Both interviewers had minimal prior contact with the participants, and the participants had no personal knowledge of the interviewers. However, the participants knew that the interviewer’s reason for doing the study was to evaluate the extended home visiting program they delivered or received. The interviews were conducted over telephone or video meeting and were held in Swedish or with the help of a translator, which was used at four occasions with parents to translate from Arabic, Italian, Amharic and Somali to Swedish. The interviews with parents were conducted around three months postpartum. No field notes were taken, and no repeated interviews were carried out. With approval from the participants, the interviews were recorded and transcribed verbatim. No transcripts were returned to the participants for comments or correction. The transcripts were reviewed in an ongoing process by the research team. After four focus groups with 11 staff members, 16 individual interviews with staff and 12 individual interviews with parents, recurring patterns were found and the collected data was considered sufficiently rich to proceed to the next step, the analysis.

### Data analysis

The data was analyzed in two steps. First the data from the parents and the staff were analyzed separately using thematic analysis with an inductive approach [22]. Then thematic network analysis was used to combine the results [23]. This process began with reading and re-reading the transcripts thoroughly, taking notes and highlighting potential interesting data. Thereafter the entire dataset was manually coded and meaning units were collated. Based on identified patterns, the initial codes were grouped into preliminary themes. The preliminary themes were reviewed, revised and re-named several times. In the process of coding and analyzing the data all authors were involved in careful considerations and joint discussions. Peer debriefing was performed as the initial findings were presented and discussed at research seminars and research team meetings where opinions and ideas contributed to further analysis. The preliminary findings were once again critically reviewed in a negative case analysis, and alternative interpretations of the material were assessed before settling for the final results. The participants did not provide feedback on the findings. As translation is an interpretive act, to avoid meaning getting lost in translation the quotes presented in the result were translated by two researchers separately then compared and agreed on for the final report.

### Ethical considerations

Ethical approval for this study was granted by The Uppsala Regional Ethics Committee (2021–02143) and informed consent was obtained from all participants prior to participating in the study.

### Rigor and reflexivity

Regarding personal reflexivity, GL is a researcher focusing on parent support and is coordinating the evaluation of ‘Together for a safe start’, of which this study is a part. She is experienced in qualitative design, brings a perspective based in anthropology and public health science and has no experience of working in a medical or clinical setting. While this research was conducted, JL was a medical student writing her master thesis supervised by GL. JL had received training in conversation methodology, had some prior knowledge of qualitative research but no practical experiences. AS is the principal investigator of this study and head of the research group. Both AS and GW has extensive experience of qualitative research. The team could capitalize on rich discussions both within team meetings and during research seminars with the broader research group.

By appreciating and validating different perspectives within the team during the discussions, the power dynamics between student–supervisor as well as researcher–research leader were not hindering an open and collaborative analysis. Regarding the differences and dynamics between researchers and participants, all staff and parents who were interviewed were being informed of the neutral stance of the researchers. We explicitly pointed out that we were from the University, not from the National Board of Health and Welfare, and that we were curious to listen to all their experiences both positive and negative. Methodological decisions were made on weekly held team meetings where all stages of the study were discussed.

Finally, regarding contextual reflexivity, the research team is part of a research group in social medicine, well versed in health inequity and eager to listen and give voice to those less heard in society. If there were any impact on the context whilst this study was conducted it may have been that some participants were positively affected by being listened to by researchers who were interested in what they had to say.

## Findings

Nine mothers, three fathers, ten CHS nurses, nine midwifes and eight social workers participated in the study; in total 12 parents and 27 staff. The parents were between 25–43 years old, with a median age of 31. Seven of them were first time parents while five had prior children. Ten of the parents lived together with a partner or co-parent while two did not. The parents were ethnically diverse: three originated from Sweden and three from Somalia, the rest were from either Ethiopia, Morocco, Tunisia, Tanzania, Alger or Eastern Europe. Four of the interviews were carried out with the help of a translator. All interviews with the parents were individual. Three parents were recruited in site A, four in site B, three in site C and two in cite D.

Regarding the staff, 16 individual interviews and four focus groups with two to three persons in each group were carried out. The age of the staff-participants ranged from 28 to 66 years, with a median age of 49. Most of them had many years of work experience in their profession and they had worked at their current clinic or family center between 0,5–16 years, with a mean of four years. Six staff members were recruited in site A, three in site B, eight in site C and ten in cite D.

**Fig. 1:**
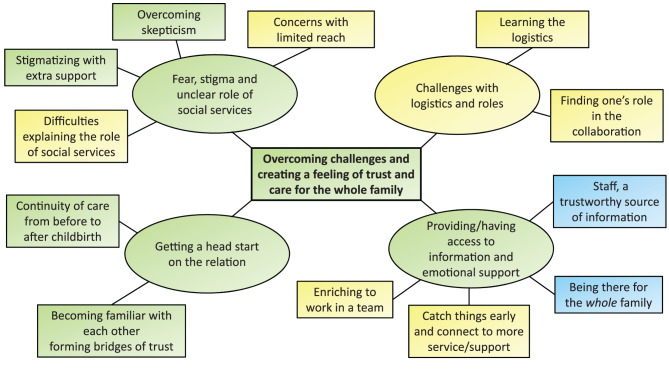
Structure of the thematic network. Themes are color coded with yellow for staff, blue for parents and green for themes shared by both staff and parents

The parents’ and staffs’ experiences as a whole are comprised in the Global Theme *Overcoming challenges and creating a feeling of trust and care for the whole family*. Which is derived from the four Organizing Themes: *Fear, stigma and unclear role of social services*; *Challenges with logistics and roles; Providing/having access to information and emotional support;* and *Getting a head start on the relatio*n. Furthermore, these Organizing Themes are derived from twelve Basic Themes (Fig. 1). The quotes presented have been assigned a number and either ‘father’, ‘mother’, ‘nurse’, ‘midwife’ or ‘social worker’ to show which of these groups the interviewee belongs to.

### Fear, stigma and unclear role of social services

The Basic Themes *Difficulties with explaining the role of Social Services, Concerns with limited reach, Overcoming skepticism* and *Stigmatizing with extra support* capture some of the challenges the staff experienced both in dealing with rumors regarding the Social Services that had spread among migrants in the communities they served and also in how to explain the role of the social worker in the program. Rumors that were prevalent in all four sites said that the Social Services “can take children away from their families”. This caused fear among some parents who were afraid of being surveyed instead of supported by the Social Services and it was a reason for families declining or being hesitant to receive the home-visits. The staff was therefore concerned that they did not reach some of the families who might benefit the most from the program. Furthermore, the use of translators when meeting parents who did not speak Swedish, made it even more difficult to explain the role of the social services.

*We have thought a lot about what word to use, how we shall express ourselves … What does the translator say when there is no word that correspond to the Swedish word? I try to explain that I work with families who need support, and already there it can go wrong when I say support because one may feel that “I don’t need support” so, it’s really difficult.”* 7, social worker

This quote also indicates the staffs’ challenges regarding offering the program in a way that didn’t make the parents feel stigmatized and question why they were being offered the home visiting program. It was quite common for the parents to wonder about the purpose, how they had been selected and if it had something to do with them personally.

*As you call yourself a social advisor, it can sound a bit like, that there is something negative about it, that we somehow need to have control over the family and that we think that they need extra support, so we have received a few such cases now where parents have felt offended and so on. And then there was a rumor that it is only selected here, and that it is only the foreign-born here, who need support, who should be part of this. So, we have a little difficulty there right now.* 14, nurse

In some cases, the antenatal visit had not been possible to carry out and the nurse and social worker met the parents for the first time at the home-visit after the child had been born. This made it more difficult to create trust and, in the case quoted below, the family did not want them to come to their home again, but preferred only to meet the nurse at the CHS clinic.

*He felt like scared of us or something, or maybe most of me since I come from the Social Services … and it did not feel so good (…) It felt like a failure I can say … Because I think that I really would have been good for this family (…) it would have been very good to have gone through the midwife, so that they would have had time to think it through. Because they were very suspicious.* 6, social worker

However, meeting the staff in person during the antenatal visit and receiving information about the program as well as having an opportunity to ask questions and unravel misconceptions often gave the parents a better understanding of the program. Understanding more of what to expect onwards led to the parents feeling reassured and safe.

*We got scared first when they said “they will meet you!”. No, why? And then I was wondering if I had done something wrong or if I couldn’t do it or if my midwife had noticed something about me… But then after I had met them, they had been introduced, well I, I got happy! Now there is someone, well someone like my mother that will help!* 25, mother

### Challenges with logistics and roles

Inadequate resources for logistics and administration were problematic in a couple of the sites during the implementation of the project. Many expressed frustration at being assigned responsibilities for scheduling and coordination without being allocated time for these tasks. An increased administrative burden took time, energy and focus from the core activities and led to stress, reduced commitment and, in the worst case, risk of burnout.

*I have almost “hit the wall”, although I have made it through. It is serious. It’s like, it’s not good. You have to, we have to be given the conditions first … This is great, but it feels like… it has to be connected to reality and that one sees this. That is, how one makes the schedule right and everything from the beginning. Because there are some who struggle.* 8, midwife

Initially, there were also uncertainties regarding the interprofessional collaboration and the distribution of responsibilities. Sometimes it was unclear who carried the main responsibility for the antenatal visit and which roles they were supposed to assume during the meeting. Furthermore, it took time and experience to get to know each other and work together as a team. Uncertainty in the collaboration could be conveyed to the parents and works against the feeling of security that one wanted to achieve.

*We don’t have time set aside for this, but this is CHS’s project and we were only supposed to help with handovers. That’s how it was presented from the beginning, and then I think a lot of material was emailed out, but I wonder if any midwives have read through this material. Because it was not our project. And then we’ve had a hard time… finding our role, and we think the CHS nurses and social workers have a hard time finding their role and we only had supervision last week together. And it’s six months after this started.* 21, midwife

The way in which the CHS and maternal healthcare were organized were of great importance to how well the coordination worked. Having co-located the CHS with maternal healthcare in a family center facilitated cooperation both relationally and logistically.

*Since there are many midwives and we are many CHS nurses, it feels like things haven’t really flowed well in these visits. You don’t really know who is in charge and who will ah, who will guide. It’s been a bit uncertain … We sit in different houses, in different, completely different sides of town. So, if you had sat and worked together and gotten to know each other better, I think you would have gained a lot from that.* 13, nurse

### Providing/Having access to information and emotional support

Almost all parents spoke of receiving information from the staff as important and highly appreciated, as captured in the basic theme *Staff, a trustworthy source of information*. They perceived the staff as experienced, professional and knowledgeable. The type of information requested and received varied between parents. It could be information about the program, the Swedish system and practical things. Many participants considered it essential that the staff had the time to listen to their thoughts and concerns, as well as answering their questions, since the information was useful and contributed to them feeling that they were cared for by the staff. In general, the parents were especially pleased with the opportunity to ask questions and discuss topics they were particularly interested in. The staff also expressed appreciation with the flexibility built into the manual, giving room to tailor the visit to different needs.

*It was great, especially considering that it was our first child, because you have a million questions that you wonder about. […] This particular combination of having a social worker and a child health nurse also makes a huge difference, because they have such broad knowledge.* 23, father

Among the participating parents, more than half originated from outside of Sweden, some having quite recently moved to Sweden. For the parents who were new in the country, the antenatal visit provided an opportunity to acquire important information about the Swedish society, such as different Swedish authorities, as well as insights about public resources and benefits.

*It was important for me to meet the nurse because my other two children were born in Africa and there’s a new system here in Sweden and we got, I got information about how everything works and what health care systems we have here in Sweden.* 30, father

Parents with limited knowledge about the Swedish society experienced that receiving this type of information gave them useful insights as well as an increased sense of security. One parent gave an example of the important knowledge she had received by explaining she would now know what to do and who to contact if she ever witnessed a mother or child being in distress. A couple of the fathers pointed out that this made them feel more at ease and prepared for the birth of the child.

Most parents mentioned an appreciation for being given both general and specific information concerning practical matters. The desired information was rather individual and ranged from learning how the baby is given their social security number, to how the co-parent best supports the mother after delivery. Many parents spoke positively about the fact that questions felt encouraged and that they experienced being able to ask about anything they had in mind. A general feeling of trust is implied by several parents, contributing to the environment in which they felt enabled to ask questions.

*What was so positive was that there were no questions that were weird and I thought that was really nice of them, that they really “Yes, but it is you who are allowed to ask questions”. So, we did ask a lot of questions and I really felt that there was, like, a trust in them.* 24, mother

Some parents also mentioned feeling confident with the staff’s education and appreciated the fact that the advice given was coming from a trustworthy source. One parent expressed feeling reassured by having the possibility to verify information and advice coming from other sources with the professional staff at the antenatal visit. Receiving information from a trustworthy source led to feelings of security for the parents as they perceived they had been given advice they could rely on. These experiences among the parents are echoed by the staff, who appreciated the flexibility of the manual, enabling them to adapt the conversations to different families.

Many parents experienced that the staff cared for them, in addition to caring for the baby. In some interviews this was directly expressed while in others it was implied. This is captured in the basic theme *Being there for the whole family*. For those mentioning it, this feeling of being cared for was highly valued.

*Yes, well I was also happy that they take care of, not only the baby, but me too! So, they will check if I need help later. Especially when it’s the first baby, if I will be sad with the baby, if I will not feel well, I will contact them and they will help me.* 29, mother

A couple of the parents mentioned their appreciation for how the visit provided an opportunity to reflect on matters such as emotions, roles and relationships.

*And I felt that I liked that feeling at the family center that like, it was not only the medical stuff, but we also talked about feelings and expectations and the role of the mother and the role of the father and our relationship to each other and stuff like that. So, I felt even then that it was… Yes, that it felt good.* 22, mother

Having the opportunity to talk about the forthcoming baby in a broader perspective than just the medical aspect was positive. A few parents found it valuable to discuss equal parenting, such as the role of the mother, versus the role of the father and the involvement of both parents. One mother found it especially useful to be able to do so with her partner in company of the staff, where the professionals were able to contribute with other perspectives.

Furthermore, the possibility of having someone to reach out to should they need to, for practical and emotional support, increased the parents’ feelings of safety and support. Especially for parents without family or friends to lean on, but also for parents with a sufficient social network.

*Psychological support, that it felt safe, that there were people who were there for me. Since I am very lonely and don’t have any relatives or anyone here, it felt very nice that I had someone who gave me extra support and was there for me.* 31, mother

The basic theme *Catch things early and connect to more service/support* is derived from staffs’ appreciation that the home visiting program provides a unique opportunity for the social services to get involved early in a child’s life and work preventively. Without this early intervention, social services often get involved too late and find it difficult to break already established habits and patterns.

*I feel that this is such a great benefit for us who work as social workers. That we get a door into people’s homes, where we can just tell them that this exists. Usually, we work with parents with much older children, about setting boundaries, etc. these are quite old children. Many things are quite tough. We come in too late, I think, far too late.* 12, social worker

*Enriching to work in a team* is the last basic theme in this organizing theme, and it captures the staffs’ experiences of learning from each other. Learning from each other’s areas of expertise as well as each other’s conversation skills led to new perspectives and inspiration.

### Getting a head start on the relation

The organizing theme *Getting a head start on the relation* is derived from two basic themes. To begin with, the basic theme *Becoming familiar with each other builds bridges of trust* illustrates how both parents and the home-visiting team experienced that meeting each other at the antenatal visit made the first home visit feel more natural. In many cases it made parents feel more secure and comfortable with the program. Others described the thought of not having the antenatal visit before the home visit as odd, expressing it would feel strange not knowing who would be coming to your home. For some others it was not as important to become familiar with the staff antenatally, these parents thought that they would still feel comfortable and would probably get to know the staff just as well in without the antenatal visit.

*It simply becomes a security and that you know that when the baby arrives, you know that they are the ones you will be in contact with and the ones that will be coming to our home. You have had the opportunity to meet them before, so that it doesn’t get, well, something strange. It becomes very natural. So, a bit of that feeling you carried with you, a feeling of security.* 25, mother

Moreover, as experienced by the staff, the midwife and the mother have already developed a relationship at the time of the visit, and the midwife often represents security for the mother. As the midwife is involved in introducing the mother to the CHS nurse and social worker, the already established relationship can “spill over” into the new relationships, which facilitates relationship building.

*I also think it may be that we have built up a relationship and trust between the patients. And when we make the handover, in some way we also approve the CHS and say that now you have been to see me and you know me and now you get to meet these people and these are good people, it will be good. We are in some way blessing it.* 17, midwife

Similarly, for some parents, having the midwife present was helpful in establishing a constructive relationship with the new staff. It was described that having the midwife present at the antenatal visit was like as a bridge, where the midwife was able to hand over to the new staff in person instead of being referring to two unknown people.

*It was also a very good meeting because then our, or my midwife was also there. (…) It became like a nice, what should one call it, a nice bridge – that the midwife could hand over something (…) I thought this was very positive, that it’s just not “Well, but now I’m leaving you and then you’ll be meeting with… those two” … It was very nice that we all could meet, like all there, in the same place.* 24, mother

According to the staff, establishing a relationship before birth helps build trust between parents and staff, which in turn acts as a lever to get more out of the program as a whole. The threshold for initiating deeper conversations and raising more personal questions and concerns is lowered, and parents can receive support for problems that would otherwise not have surfaced. Having already passed the ‘getting to know you’ stage also makes it possible to make better use of the first home visit: The atmosphere is more relaxed, it’s possible to refer back to previous conversations, and because staff have a better understanding of the parents and their background, they are better able to provide more tailored support.

The second basic theme: *Continuity of care from before to after childbirth* elaborates further on how the antenatal visit increases the continuity of care, which benefits both parents and staff. The visit is an opportunity for the CHS nurse to obtain information about the mother together with the midwife (with the mother’s consent) that would otherwise be protected by confidentiality. This makes it possible to follow up on any challenges during pregnancy at later home visits.

*They have already shared some information with us that may also be very important ahead of the first visit. Are they single, is it IVF, or has there been several miscarriages before, is it an unplanned pregnancy, is it very much wanted? Have there been problems at home that we need to know about? In a way, we have already talked about their social situation […] They can feel secure […] It’s not like “I have to get to know a new person again and now I have to tell them everything all over again”.* 14, CHC nurse

Continuity can also be satisfying for staff – seeing where the family goes next gives a good ending for the midwife, and coming in during pregnancy and following the family’s life journey increases work satisfaction for CHS nurses and social workers.

Furthermore, according to the staff, the antenatal visit helps parents to reflect on the time *after* the birth not just the birth itself. It allows the expectant father/co-parent to be included during a period that is otherwise strongly focused on the mother and pregnancy. Involving the expectant father and giving him space to raise his concerns and ideas early on provides the opportunity to awake more curiosity for the child and to confirm and strengthen the father in his role as a parent. In combination with discussing issues regarding the division of responsibilities early on, this creates good conditions for working towards equal parenting.

## Discussion

### Main findings

The aim of this study was to gain a deeper understanding of the parents’ and staffs’ experiences regarding an antenatal visit at the maternal health care center, which had been added to an extended home visiting program in order to introduce the staff that would carry out the home visits once the child was born, as well as discuss some topics and give room for the expectant parents’ own questions. The findings show that this visit played an important role in overcoming challenges regarding parents’ distrust of the social services and it created a feeling of security and care for the whole family. Both parents and staff expressed that the meeting enabled trust to spill over from the familiar midwife to the unfamiliar CHS nurse and social worker, which gave the relationship between the staff and the parents a head start. This made the first home visit (once the child was born) more relaxed and enabled conversations with more depth as well as more tailored support.

Since the program is implemented in disadvantaged areas, many parents face different kinds of struggles related to low socioeconomic status and consequently have a need for support that goes beyond parental support. The findings show that the antenatal visit gave staff more understanding of what specific support needs each family might have and that they could adjust the topics to discuss accordingly. For example, some parents who were newly arrived immigrants obtained valuable information about the Swedish system at the antenatal visit. Having the opportunity to raise their own questions and concerns during the visit was appreciated by the parents.

### Interpretation

These findings resonate with earlier studies. The Organizing Theme *Fear, stigma and unclear role of social services*; highlight the importance of being able to clearly describe the purpose of the program and especially the role of the social worker. If this is not clear to begin with parents may have reservations, which is similar to what Rautio found regarding parents’ experiences of receiving early support and home visits in Finland [24]. Another similarity with Rautio’s work is the importance of familiarity as conveyed in the organizing theme *Getting a head start on the relation*. This organizing theme highlights the role the antenatal visit play for parents and staff to become familiar with each other and is consistent with Mangrio and Hjortsjö’s findings that being familiar with each other facilitates conversations where parents feel free to express their concerns [25]. The positive aspect of having an established relationship with the midwife who already “have the whole picture of the family” has also been observed by Sjögren Forss et al. [26]

The Organizing Theme *Providing/having access to information and emotional support* show that the antenatal visit is carried out in accordance with Barboza et al.’s conceptual model of a situation-based practice, where families’ different support needs and situations are taken into account [16]. This theme also resembles findings from Tiitinen Mekhail regarding parents’ appreciation of gaining more knowledge of local resources and societal services [17]. It is also consistent with findings from Sjögren Forss et al. regarding parents’ trust in the professionals and appreciation of receiving information from the multiprofessional team [26]. That parents appreciate being given the time to raise their own questions or concerns has also been pointed out by Bäckström et. al. [27]. Furthermore, we found that being able to provide such support and better meet the needs of children and families by working in a multiprofessional team was highly appreciated by the staff, a finding echoed by Nygren et al. [18] as well as by Mangrio and Hjortsjö [28], Golsäter and Andersson [29] and Larsson et al. [30]. Golsäter and Andersson’s as well as Larsson et al.’s findings are also similar in lifting the staff’s experiences of being able to better create caring relationships with the families and better tailor support to their needs.

Just as in Mangrio and Hjortsjö’s findings, the theme *Challenges with logistics and roles* shows that the implementation of a multiprofessional home visiting program demands resources, not only time for providing the visits but also for planning the logistics, and that it takes some time for the team to find their roles in the collaboration [28]. The value of having co-located services, as in a family center, facilitating the interprofessional collaboration and the logistics has also been highlighted by Golsäter and Andersson [29].

### Strengths and limitations of the work

Regarding the sample, it is a strength that all involved staff were interviewed; including all three professional categories from all four pilot sites. However, there is a risk of selection bias when it comes to the sample of participating parents. Even though staff were encouraged to ask all parents who had been to the antenatal visit it is unclear if they did so, or on what basis any selection was done. It is also unknown how many parents in total were asked and how many declined, and why. It is not unreasonable to assume that parents displeased with the home visiting program and/or the antenatal visit, were less willing to participate. Also, the midwives and CHS nurses might have been less prone to ask a displeased parent. Additionally, we found it harder to recruit fathers and only ended up interviewing three. If they declined to participate to a greater extent, or were generally less asked than the mothers, is unknown. In case this affected our findings is uncertain but should be considered as a potential threat to the transferability of the study.

Including parents who do not speak Swedish and using an interpreter made it possible to interview parents who would otherwise not be heard. Even though the quality of the data may be a little poorer when using an interpreter, it gave more variation to the sample.

Some of the participants were interviewed quite a long time after the antenatal visit, which can have resulted in recall bias. We reasoned that a desirable time to conduct the interviews would be around two months postpartum, when the parents had had a couple of home visits after birth and still likely would remember the antenatal visit. This was the case for most of our participating parents. However, due to a slow recruitment rate, we did interview parents up to six months postpartum, in order to include as many parents as possible. This led to the benefit of more data, in terms of more participants. It also led to some limitations, for example that some participants did not remember the antenatal visit all that well or were confusing the antenatal visit with other visits. It is also possible that matters which felt important to a parent during the visit or close after, no longer feel as relevant long after and therefore were not mentioned.

Since the whole society was affected by the Covid-19 pandemic during the time of the study, it influenced the parents’ frames of reference which in turn could have affected how the parents experienced the antenatal visit. For example, co-parents were rarely allowed to accompany the pregnant parents at their usual maternal health visits and group activities for expectant parents were cancelled. These circumstances might have made the antenatal visit more appreciated by the parents.

Finally, the research team have various backgrounds and levels of experience both with clinical work and with qualitative research and could draw from these different perspectives during the analysis.

### Recommendations for future research

Extended home visiting programs have a long tradition in countries outside Scandinavia. In the Scandinavian welfare states, however, universal child health services have been routine practice for decades, reducing the need of specialized services for vulnerable families. Nevertheless, with globalization and a changing society in Sweden the needs of families have become more diverse. Therefore, further research on extended home visiting programs, their broader acceptability, uptake, effectiveness, and sustainability is needed.

### Implications for policy and practice

The antenatal visit plays an important role in building parents’ trust for the child health care nurses and social workers providing the extended home visiting program. This visit can have a positive impact on the reach of such services and create more continuity of care and sense of safety for parents through the transition to parenthood.

## Conclusion

Building trust and creating a sense of security are important aspects of what is means to introduce a home visiting program and the staff who will provide it, during an antenatal visit led by a midwife. Both parents and staff noted that it facilitated trust transfer from the midwife to the CHS nurse and social worker. Getting a head start on the relation was benefiting future home visits by enabling more relaxed and in-depth conversations. The visit also helped staff identify families’ specific needs and tailor the support accordingly. However, adequate resources for logistics and administration are a prerequisite for implementing a common visit between agencies.

## Electronic supplementary material

Below is the link to the electronic supplementary material.

Supplementary Material 1

Supplementary Material 2

## Data Availability

Pseudonymized transcripts of the interviews can be shared upon request.

## Declarations

### Reporting method

This report follows the Consolidated criteria for reporting qualitative research (COREQ).
