# Supplementary material for: A safe bridge – parents’ and staff’s experiences of an antenatal visit introducing a home visiting program in disadvantaged areas
Source: BMC Health Serv Res. 2025 Oct 22;25:1392. doi: 10.1186/s12913-025-13578-9 (PMC12542035; doi:10.1186/s12913-025-13578-9)
Supplement: Supplementary file 1 — Supplementary Material 1 [file 12913_2025_13578_MOESM1_ESM.docx]

**Interviewguide 1 – Parents**who participated in the first visit of *Together for a safe start*

*Semi-structured interviews, which start with an introductory question and continue with probes to guide the conversation.*

To begin with, can you tell me how old you are and if this is your first child or if you have more? How long have you lived here in (name of area + in Sweden if born abroad + country of birth)?

This interview will be about your experiences of the visit at the maternity care center, where you also got to meet the CHC nurse and the family counsellor. Can you tell us about how this visit was for you?

How important was it to you that you were able to meet the CHC nurse and the family counselor already during the pregnancy?

Was there anything you talked about during the visit that you found (extra) interesting or helpful?

*Headings from guidance materials: The child, Parents, Paying attention, Information about the extended support, Becoming parents, Support and feeling well*

Was there anything the staff brought up that you didn't find helpful or uninteresting? (Can you tell me about what if so)

Is there anything you wish you had talked about that you didn't? (Can you tell me about what...)

Was there anything you talked about during this visit that made you more curious about your child and what your child can do, even now? (Please give examples)

Was the child's father (or partner/co-parent) present at the visit? Did you talk about participation and equal parenting, if so, how was that for you?

Do you have any suggestions for improving this way of working?

Is there anything else you can think of, anything you want to add?
