# Supplementary material for: A safe bridge – parents’ and staff’s experiences of an antenatal visit introducing a home visiting program in disadvantaged areas
Source: BMC Health Serv Res. 2025 Oct 22;25:1392. doi: 10.1186/s12913-025-13578-9 (PMC12542035; doi:10.1186/s12913-025-13578-9)
Supplement: Supplementary file 2 — Supplementary Material 2 [file 12913_2025_13578_MOESM2_ESM.docx]

**Interviewguide 1 – Staff**

CHC nurses, midwives and family counsellors, who have started working with the first visit in Together for a safe start.

This interview will be about your experiences of working with the first visit in Together for a safe start. First, I would like to know how old you are and what experience you have as a midwife/nurse/family counsellor?

To begin with, can you tell us about how you carry out the visit now? (covid adaptation)

Now that you've resolved it this way, how do you think it would be if you could carry out the visit as originally intended? If you could compare it.

What do you think is the intention of this first visit?

How does it feel to follow the guidance material and carry out the visit?

Is something working better or worse during the visit?

*Headings from guidance materials: The child, Parents, Paying attention, Information about the extended support, Becoming parents, Support and feeling well.*

If there are any adjustments made to the content, can you describe them?

To work together to offer better continuity and security in care chains and create a relationship with CHC nurses and family counselors. How do you think it works?

The thing about awaking curiosity about the child's competencies, can you describe how you think it works? Please tell us about an example of when it was successful and when it was not.

Raising the participation of fathers or co-parents and equal parenting, how does it work on the first visit do you think?

Regarding families in the target group who choose not to participate, what do you think is the reason?

Have you encountered any obstacles, if so what?

Please describe if you think you get enough support in the material or if something more is needed to strengthen your competence in this task. What then?

What suggestions for improvements do you have?

Is there anything else you can think of, anything you want to add?
